# Community structure informs species geographic distributions

**DOI:** 10.1371/journal.pone.0197877

**Published:** 2018-05-23

**Authors:** Alicia Montesinos-Navarro, Alba Estrada, Xavier Font, Miguel G. Matias, Catarina Meireles, Manuel Mendoza, Joao P. Honrado, Hari D. Prasad, Joana R. Vicente, Regan Early

**Affiliations:** 1 InBIO/CIBIO - Centro de Investigação em Biodiversidade e Recursos Genéticos, Universidade de Évora, Évora, Portugal; 2 Spanish Scientific Council (CSIC), Centro de Investigaciones sobre Desertificación (CIDE, CSIC-UV-GV), Moncada, Valencia, Spain; 3 Research Unit of Biodiversity (UMIB, UO-CSIC-PA), Oviedo University – Campus Mieres, Spain; 4 Departament de Biologia Vegetal, Facultat de Biologia, Universitat de Barcelona, Barcelona, España; 5 Imperial College London, Ascot, Berks, United Kingdom; 6 Spanish Scientific Council (CSIC), National Museum of Natural History (MNCN), Department of Biogeography and Global Change, Madrid, Spain; 7 InBIO / CIBIO - Centro de Investigação em Biodiversidade e Recursos Genéticos, Campus Agrário de Vairão, Universidade do Porto, Vairão, Portugal; 8 Faculdade de Ciências da Universidade do Porto, Edifício FC4 (Biologia), Porto, Portugal; 9 Physical Sciences and Engineering Division, King Abdullah University of Science and Technology, Thuwal, Saudi Arabia; 10 Centre for Ecology and Conservation, College of Life and Environmental Sciences, University of Exeter, Cornwall Campus, Cornwall, United Kingdom; Universita degli Studi della Tuscia, ITALY

## Abstract

Understanding what determines species’ geographic distributions is crucial for assessing global change threats to biodiversity. Measuring limits on distributions is usually, and necessarily, done with data at large geographic extents and coarse spatial resolution. However, survival of individuals is determined by processes that happen at small spatial scales. The relative abundance of coexisting species (i.e. ‘community structure’) reflects assembly processes occurring at small scales, and are often available for relatively extensive areas, so could be useful for explaining species distributions. We demonstrate that Bayesian Network Inference (BNI) can overcome several challenges to including community structure into studies of species distributions, despite having been little used to date. We hypothesized that the relative abundance of coexisting species can improve predictions of species distributions. In 1570 assemblages of 68 Mediterranean woody plant species we used BNI to incorporate community structure into Species Distribution Models (SDMs), alongside environmental information. Information on species associations improved SDM predictions of community structure and species distributions moderately, though for some habitat specialists the deviance explained increased by up to 15%. We demonstrate that most species associations (95%) were positive and occurred between species with ecologically similar traits. This suggests that SDM improvement could be because species co-occurrences are a proxy for local ecological processes. Our study shows that Bayesian Networks, when interpreted carefully, can be used to include local conditions into measurements of species’ large-scale distributions, and this information can improve the predictions of species distributions.

## Introduction

Current topics in ecology such as biological invasions or species responses to global change rely on a better understanding of the drivers governing species distributions [1,2]. Although at large geographical scales climatic conditions are the main factor determining species distributions (but see [3]), several studies have shown that non-climatic biotic and abiotic factors (e.g. landscape dynamics, disturbance regimes, micro-topography, biotic interactions between species such as competition or predation) are important at finer spatial resolutions [4–9]. Therefore, information reflecting local ecological processes would be valuable for improving forecasts of responses to environmental change by species distribution models (SDMs). Nevertheless this information is rarely included (but see [10]).

A potential reason why local factors are not usually included in SDMs is the lack of suitable fine scale data over large areas. Although data on micro-environmental and biotic interactions are usually not available at a large scale, for many taxa, in particular plant species, the relative abundance of coexisting species in a community is well documented across large geographic areas (e.g. in vegetation databases such as SIVIM (http://www.sivim.info/sivi/), BDN (http://www.magrama.gob.es/es/biodiversidad/servicios/banco-datos-naturaleza/), BIEN (http://bien.nceas.ucsb.edu/bien/). An additional challenge specific to biotic interactions is finding statistical techniques to deal with the large amount of potential interactions. There have been previous attempts to include biotic information into SDMs [11], one approach is to focus on a small number of pair-wise species dependencies (< 25 species) [12–15] and another to use surrogates for biotic interactions, such as species richness [16]. However, these approaches are either unable to assess all potential species interactions (there are N^2^ –N / 2 possible pair-wise interactions in a community that contains N species), or they rely on extremely detailed ecological knowledge. Finally, the statistical challenge is made much more complicated when considering that species live in complex interaction networks, where co-occurrence patterns are affected by not only pair-wise but also indirect interactions influenced by the presence of a third species [17,18].

Bayesian network inference (BNI) can be a useful tool to overcome these major challenges. These analyses are used to study the conditional dependencies (represented by directed edges) among a set of either abiotic (i.e. climatic, edaphic or land-use-related) and/or biotic (i.e. species abundances) variables (represented by nodes). BNI has been widely used to study interaction patterns in molecular biology, medical informatics, economics and social science research [19–23]. However, BNI has only been recently applied to ecological research questions: to microbial community ecology, to the study of assembly rules in invertebrate and bird species, to inform management decisions, and to disentangle direct and indirect associations between environmental variables and species distribution patterns [24–31]. BNI estimates the effect of specific interactions on a focal species considering all the potential direct and indirect relationships among the rest of species in the community. To calculate the effect of every direct and indirect interaction requires the estimation of a very high number of parameters (i.e. assigning a probability to each potential combination of states of every species). This is unfeasible using regression techniques, but is possible with BNI due to its heuristic nature. BNI uses a heuristic search of graphs proposed by different algorithms, which are sequentially compared to the dataset through goodness-of-fit statistics. The graph that best matches the relationships between variables in the data is kept [23]. In addition, BNI decomposes the global probability distribution of the abundance of a focal node (species), into a local probability distribution, only affected by a set of conditioning variables [23]. Thus, BNI can combine abiotic and biotic information, and consider the potential effects of the composition and relative abundance of every species in a community (hereafter ‘community structure’) on a focal species [23,32]. Based on this information, BNI summarizes the entire community structure by calculating the strength of the effect of ‘parent nodes’ on ‘child nodes’[23,32], and each species can be a parent or child to any other species. Larsen et al. (2012) [29] were the first to show that BNI can be combined with regression techniques to improve predictions of species’ relative abundances in a community. They suggested that BNI can be used to identify the most influential parent and child nodes for a target species. Each of these nodes (species) can be entered into SDMs, which are used to predict the target species’ distribution and resulting community structure.

Although BNI can identify patterns in species associations, it cannot disentangle the two major underlying processes shaping the relative abundance of species in a community, biotic interactions and environmental filtering [33,34]. Biotic interactions can prevent a species from occupying all areas that are environmentally suitable for it (e.g. competition, predation), but at the same time extend the distribution of a given species into areas that would be environmentally unsuitable in the absence of the biotic interaction (e.g. facilitative interactions) [35,36]. Environmental filtering restricts species distributions to sites where environmental conditions are suitable for a given species. This includes environmental conditions that vary at large spatial scales (e.g. climate or lithology), and micro-environmental factors that vary at local scales (e.g. pH, soil humidity or shade). At local scales, the presence of species with certain requirements could indicate the availability of suitable micro-environmental conditions for other species that share similar environmental requirements. Thus, the same pattern of species co-occurrence could be caused by both biotic interactions and micro-environmental filtering. As the use of co-occurrences to study biotic interactions becomes more widespread, it is important to consider how these two processes could be disentangled. A solution to this problem might lie in addressing the ecological requirements of the species involved, as indicated by species traits, and we explore how this could be done.

In this study, we hypothesized that the relative abundance of coexisting species can improve the predictions of species geographic distributions made by SDMs. For 1570 assemblages of 68 Mediterranean woody plant species, we applied (BNI) to incorporate community structure into SDMs. We assessed the accuracy of predictions of species abundance and community structure based on SDMs with and without information on coexisting species. We used species trait data to interpret the ecological processes potentially underlying the species associations inferred by BNI.

## Materials and methods

### Overview of the methodology

Following [29], we used BNI to infer a) the “overall network” (i.e. considering all species and environmental variables) and select the parent nodes of each focal species and the sign of the inter-specific association; and b) another network for each species, in which only the focal species and environmental factors were included. Then, for each species we fitted two SDMs in which the predictor variables were the parent nodes of the focal species in each of the two networks (hereafter called “Env+Bio” and “Env” predictors respectively). Next, we compared the ability of SDMs with the two predictor types to predict the abundance of each species, and the community structure of each site.

In order to explore the ecological processes underlying the inferred species associations, we classified 68 Mediterranean plant species into two groups, each consisting of similar combinations of life-history traits and ecological requirements (see *Species syndromes* below). We used a chi-square test to assess whether species with positive or negative abundance co-variance tend to be more similar (belong to the same group) or dissimilar (belong to different groups) than expected by chance.

### Study site and community structure database

Within the Iberian Peninsula (mainland Portugal and Spain) (S1 Fig), we aimed to select a pool of plant species that do not have extremely different environmental requirements, for which differences in their distributions are entirely driven by the local conditions (for example avoiding the mix of plants from alpine and saltmarsh vegetation). In order to detect effects of the local environment or biotic interactions, the study species needed to differ in subtler aspects of their niche (for example shade or soil moisture requirements). The goal was to obtain assemblages that contain many of the same species, but that have different community structure (i.e. relative abundances). In order to obtain this species pool, we selected a species with restricted habitat requirements but which is broadly distributed throughout the Iberian Peninsula, the cork-oak (*Quercus suber*), and the pool of plant species associated with it. To determine the species associated with *Q*. *suber*, we used data from the SIVIM database (Sistema de Información de la Vegetación Ibérica y Macaronésica; http://www.sivim.info/sivi/). SIVIM compiles plant community information from phytosociological relevés (hereafter ‘plots’) consisting of directly submitted data, publications, and unpublished documents (e.g. theses or reports) [37]. For each plot the species composition and relative abundance (percentage of cover) of each species was reported (more details in Methods appendix). We extracted all SIVIM plots in the Iberian Peninsula in which *Q*. *suber* was present, and the relative abundances of co-occurring species in those plots. This resulted in 1570 plots occupied by 68 plant species (S1 Table).

### Environmental variables

Each plot was characterized based on the following environmental variables: climate, geology, land use (agriculture or forest-shrub), orientation, and dominant growth-form of the vegetation (trees or shrubs). Climatic variables were obtained from a dynamical downscaling method using the Weather Research and Forecasting model [38] (more details in Methods appendix). Geological information was obtained from the digital geological map data provided by OneGeology-Europe (http://www.onegeology.org/), and each plot was assigned to the dominant geological type, i.e. that which covered ≥70% of the 10 km grid cell in which each plot was located. If no single type fulfilled this requirement, the plot was assigned to a type called “mix” (more details about geological types in Methods appendix). Land use information was extracted from the European Environment Agency website (Corine Land Cover 2006; http://www.eea.europa.eu/). We classified each 10 km UTM (Universal Transverse Mercator coordinates system) grid cell into just one of the two main land uses, agriculture and forest-shrub, based on the dominant land use type, or into a third category (mix) when neither of the land uses covered 70% of the surface. Orientation determines the solar irradiance a site receives, affecting the microclimatic conditions, and resulting in larger hydric stress in south oriented aspects. Plot orientation (North (N), South (S), East (E), West (W), North-East (NE), North-West (NW), South-East (SE), South-West (SW)) was extracted from the information included in each entry of the SIVIM database. The dominant growth form (trees or shrubs) was considered “tree” if the percent of tree cover reported for that plot was more than 50%, and “shrub” if tree cover was less than 25%. If the percentage of tree cover was between 25–50% the plot was considered a ‘mix’. In order to account for trends in the data across large geographical distances, the longitude and latitude of the grid cell in which each plot was located was also used as an environmental variable.

### Network inference

We used BNI to infer relationships between the relative abundance of the 68 plant species across the 1570 plots. BNI can identify which variables (i.e. the relative species abundance or environmental conditions in each plot) significantly condition the probability of finding a given abundance of a given species [39]. The nodes of these networks represent the variables, while the directed edges (links) show the dependency between the two variables involved. Directed edges point from parent to child nodes. As species abundance was recorded as ranges of percent cover, we used multinomial Bayesian networks, in which all the variables are categorical (see details about the criteria to define categories and selection of the algorithms to infer the network in the Methods appendix).

Milns et al (2010) pointed out that directionality in a BN is hard to assess as there are multiple configurations of the network that can equally maximize the match with the observed relationships among variables. In order to overcome this issue, we used a two-step process following Sachs et al. [40]: (i) Candidate associations among random variables were identified using the 50% cut-off. The network structure is learned 500 times and the links and directions that consistently (i.e. in > 50% of the runs) show a given direction across the 500 runs are selected). The number of runs in which a link showed the same direction was used to quantify the robustness of the direction. (ii) Significant associations were identified based on the threshold approach proposed by Scutari et al. (2013) [41]. For all significant links, we calculated the sign of the interaction using a Jonckheere trend test for ordered factors [42] (see Network inference section in Methods appendix for more details about the order of the categorical variables). We partially constrained the inference by not allowing the species abundance to influence environmental variables and by not allowing any environmental variable to influence the following variables: the temperature in the warmest quarter of the year, mean annual precipitation, geological type and orientation. All analyses were performed using the package “bnlearn” implemented in the software R version 3.1.2 [32].

### Similarity in species life-history traits and ecological requirements: Species syndromes

The plant species that currently co-exist in the Mediterranean basin are a mixture of species that originated at different times and under different environments [43]. The dry, hot summers of the Mediterranean climate originated in the late Pliocene [44]. At that time, most of the plants in the Mediterranean that required summer rain became extinct and predominantly those species with traits that confer tolerance to summer drought persisted until today [45–48]. However, other plant lineages that also currently inhabit Mediterranean areas originated more recently and have evolved under Mediterranean climate [44]. Differences in the selective pressures experienced by these two groups of Mediterranean plant lineages has resulted in different morphological-functional trait combinations and regeneration niche requirements, which we term “syndromes” [43,46]. The recent lineages (with a Quaternary syndrome) are characterized by non-sclerophyllous leaves, facultative summer deciduousness, hermaphroditic, large, colored flowers, small seeds and pollination by large insects. Ancient lineages (with a Tertiary syndrome) are evergreen plants with sclerophyllous leaves, reduced-greenish-unisexual flowers, medium to large seeds, fleshy fruits dispersed by vertebrates, and pollination by wind or small insects [49].

Most of the plant species considered in this study (60 out of 68) belong to genera that have been previously assigned to one of these two syndromes according to the outcome of a principal component analysis based on their ecological traits and regeneration niche requirements [43,46] (33 as Tertiary (T) and 27 as Quaternary (Q); S1 Table). We therefore restricted this part of the analysis to those 60 species. We used a χ^2^ test to assess whether positive abundance covariance between species that have similar (the same syndrome) or dissimilar (different syndromes) ecological requirements occur more frequently than expected by chance.

### Species distribution models

We fitted SDMs to each of the 68 species. Following Larsen et al. (2012) [29], we used the network structure learned using BNI to identify the parent nodes of each species and used those nodes as explanatory variables. We used the mean percent of cover of each species in each plot as the dependent variable to construct a generalized additive model (GAM) with a binomial error distribution, including the longitude and latitude interaction of the 10 km grid cell as a smoothing term [50–52]. Cross-validation was used to estimate the optimal amount of smoothing (λ). During cross-validation, the optimal λ, and the effective degrees of freedom was obtained by choosing different values of λ and then minimizing the sum of squares of the linear regression penalized by the smoothing splines. This was performed using “mgcv” package implemented in the software R version 3.1.2 (Wood 2011). We fitted the GAMs using all the parent nodes of each focal species identified by BNI (usually 1–4 variables per species, S2 Table). If the species did not have any parent node, the GAM was fitted using the intercept as the only explanatory variable (indicated as ~ 1, in S2 Table). For longitude, latitude, mean temperature of warmest quarter and annual mean precipitation we used continuous data in the GAMs. As we aim to compare predictions made with the best available information on the drivers of each species distribution in the presence and absence of species co-occurrence data, the environmental predictors may differ between Env and Env+Bio SDMs for a given species (S2 Table). Finally, we also asked whether the models used following this procedure predicted the observed abundances better than the models based on randomly selected variables (Methods in appendix).

### Comparing SDMs with “Env+Bio” and”Env” variables

Following Larsen et al. (2012) [29], for each species we fitted two models using “Env+Bio” and “Env” predictor variables separately. To identify “Env+Bio” variables we inferred a single BN considering all species relative abundances and environmental variables, so that either species or environmental variables could be parent nodes of the focal species. For “Env” variables, we inferred network structure for each species, which contained the focal species’ relative abundance and all the environmental variables. In this way, the parent nodes of each species could only be environmental variables.

The two sets of predictor variables represent different knowledge situations. ‘Env’ asks which environmental variables we would think are important if we knew nothing about co-occurring species. Env+Bio asks which environmental variables and species co-occurrences are important when we have knowledge of both of these factors.

In order to evaluate the explanatory power of the SDMs with and without biotic data, we randomly selected two thirds of the plots in which each species was present to construct GAMs with the two sets of relevant explanatory variables (‘calibration plots’). The same plots were used to evaluate SDMs with and without biotic data. In order to account for variation in the number of explanatory variables used in “Env+Bio” and “Env” models, we calculated the Akaike Information Criterion (AIC) of each model, which penalizes against the addition of explanatory variables. We compared AICs between models using a paired t-test. We also calculated the percentage of variance explained by the two GAMs as a proxy for the absolute quality of the models. The analyses were performed using the R package “MASS” and “mgcv” implemented in the software R version 3.1.2 [53,54].

In order to evaluate the predictive power of SDMs, we used the GAMs constructed with calibration plots to predict the community structure (species composition and abundance) in the remaining one third of the plots (‘validation plots’). We calculated the Spearman correlation coefficient (rho) between the observed species abundance and the abundances predicted by the “Env+Bio” and “Env” predictors. A paired t-test on the rho values was used to test whether the predictions by the GAMs using “Env+Bio” or “Env” predictors correlate better with the observed abundances. Finally, we used the Bray-Curtis (BC) dissimilarity index to estimate the similarity between the predicted and observed community structure in each of the validation plots. Hereafter we will use the similarity index 1-BC (where 1 is the most similar, implying better predictions and 0 the most dissimilar and implying worse predictions) and refer it as “BC similarity index”. A paired t-test was used to test whether the BC similarity index was higher when the “Env+Bio” or “Env” predictors were used. These analyses were performed using the R package “vegan” implemented in the software R version 3.1.2 [55].

## Results

### Overall BNI network

**Fig 1:**
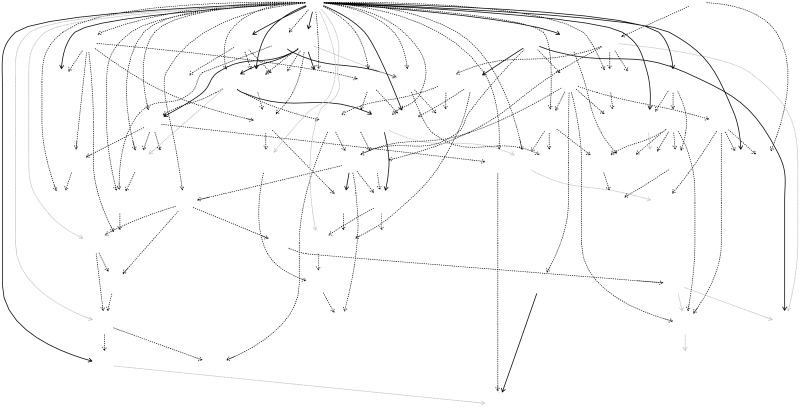
Network structure learned using Bayesian network inference. Only significant links are presented, and grey lines indicating links with no sign was detected. Grey and black circles represent species with a Quaternary and Tertiary syndrome respectively. White circles are either environmental variables (mean temperature in the warmest quarter of the year (Twarm), annual precipitation (anualP), soil types (soil), land use (landuse), orientation (orientation), dominant form (dom_form) and spatial location (spac)) or species with no syndrome associated. Continuous and dashed lines represent negative and positive associations respectively. Complete names for species are provided in the appendix and environmental variable categories in the methods section.

The overall network, including all species and environmental variables, contained a total of 138 significant links (Fig 1), 104 of which were positive (75%) and 20 (15%) negative. For 14 links the Jonckheere trend was not strong enough to assign a sign. Of the 138 significant links, 75 occurred between species. Most species-species links (95%) were positive, indicating that the probability of finding a higher abundance of one species increases when the other species is also abundant. Only four links between species were negative (S3 Table). On average, each species had 1.94 ± 0.08 (mean ± SE) parent nodes and 1.29 ± 0.18 children nodes.

### The accuracy of SDMs when informed by community structure

Across all species, the “Env+Bio” predictors resulted in models of species abundance that have greater explanatory power than did the “Env” predictors (mean (±SE) decrement in AIC = -146 ± 100; t_paired_ = -3.97, df = 67, p-value < 0.0001) (S1 Table). Across all species, the models of species abundance using “Env+Bio” predictors explains a slight but significantly higher percentage of deviance than the models using “Env” predictors, (mean increment in the percentage of deviance explained (±SE) = 1.5% ± 0.42; t_paired_ = 3.54, df = 67, p-value< 0.001), but there was considerable variation across species, ranging from species for which the model using “Env+Bio” predictors decreased the deviance explained by 6% (*Pterospartum tridentatum*) to species in which the model using “Env+Bio” predictors increased the deviance explained by 15% (*Salix atrocinerea*). The models using “Env+Bio” predictors also predicted the observed abundances better than the models based on randomly selected variables; on average, Env+Bio predictors explained a higher percentage of deviance (3.18% ± 1.29; t_paired_ = 2.36, df = 67, p-value = 0.01) (more details in Methods in appendix).

**Fig 2:**
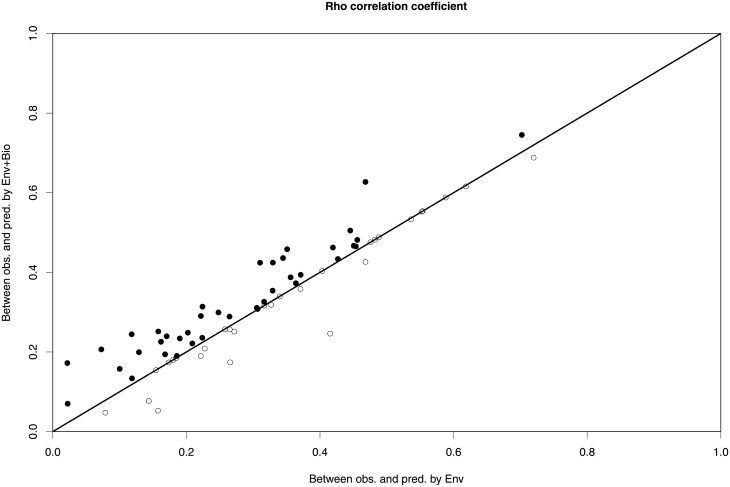
Correlation between the prediction of “Env” and “Env + Bio” models. Rho coefficient of the correlation between the observed species abundances and abundances predicted by “Env+Bio” vs. the correlation coefficient between abundances observed and predicted by “Env” models, for the 68 species. Black points above the line represent species with higher Spearman’s rho correlation coefficients values using “Env+Bio” rather than “Env” predictors. The opposite is true for white points below the line.

**Fig 3:**
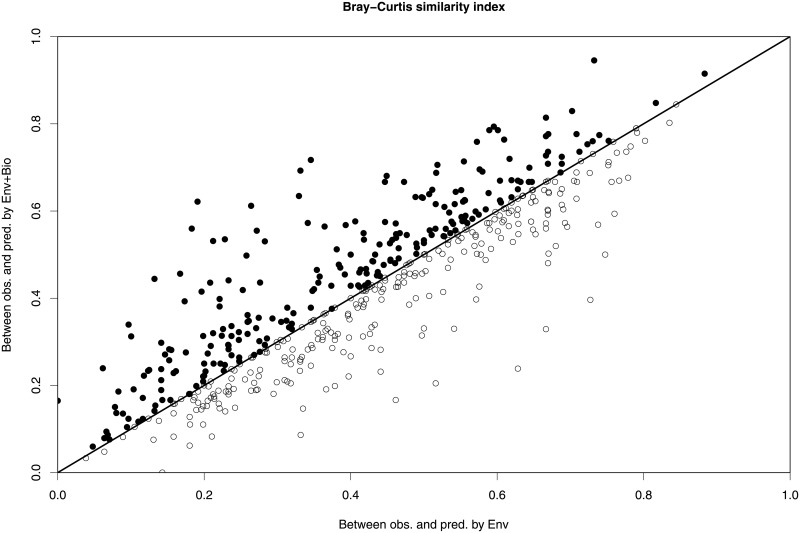
Correlation between the Bray-Curtis similarity index using “Env” and “Env + Bio models”. Bray-Curtis similarity index between the observed community structure and the community structure predicted by “Env+Bio” vs. the similarity index between the observed community structure and that predicted by “Env” models, for the 524 validation plots. Values of Bray-Curtis similarity index closer to 1 imply that community structure is predicted more accurately and values closer to 0 indicate less accurate predictions. Black points above the line represent plots with higher similarity between the observed values and those predicted using the “Env+Bio” rather than the “Env” predictors. The opposite is true for white points below the line.

Including community structure in SDMs improved the accuracy of the species’ observed abundance predictions, as there was a slight but significant higher correlation between the observed and the predicted abundances using “Env+Bio” predictors than using “Env” predictors (mean increment in rho (±SE) = 0.02 ± 0.006; t_paired_ = -3.1, df = 67, p-value < 0.002) (Fig 2). However, there were six species for which the models using “Env+Bio” predictors resulted in an increment of the Spearman correlation coefficient above 0.10, indicating a considerably more accurate prediction of these species’ abundances (S2 Table). Models using “Env+Bio” predictors also improved the predictions of the whole community structure in each plot. Overall, the Bray-Curtis similarity index was higher when using “Env+Bio” predictors than when using “Env” predictors (mean increment in Bray-Curtis similarity index (±SE) = 0.1 ± 0.004; N = 524; t_paired_ = 2.1861, df = 523, p-value < 0.0001) (Fig 3).

### Potential ecological processes underlying abundance covariance between species

The links between species inferred by BNI do not occur between random pairs of species. Positive links between species with the same syndrome (Tertiary-Tertiary (TT) or Quaternary-Quaternary (QQ)) are significantly more frequent than expected by chance (χ2 = 26.68, df = 1, p-value < 0.0001). The links were significantly more frequent between species with the same syndrome than between species with a different syndrome (Number of links: QQ = 20, TT = 32, QT = 4, TQ = 7; χ2 = 63, df = 3, p-value < 0.0001), and especially between those sharing a Tertiary syndrome (S3 Table). Only four of the significant links were negative, which prevented us from performing any statistical inference for negative links.

## Discussion

For 80% of the 68 species, including information on community structure in SDMs appears to improve predictions of species distributions. The improvements in SDM performance are of a similar magnitude to those recently found by [56], who used BNs to directly model biotic interactions and shared habitat requirements’ relationships among species in a community. Positive associations between Mediterranean woody plants tend to occur between ecologically similar (i.e. ‘Tertiary’) species. This association pattern suggests that positive associations might be driven by a match between the requirements of similar species and the presence of environmental conditions, in particular shade and moisture. The species associations we observe appear to reflect the conditions that occur within vegetation plots, and so at a much finer spatial resolution than is usually possible to study with most sources of climate data. Moreover, we selected a study system in which the macro-climatic conditions did not vary greatly. Thus, we propose that species distribution predictions might have been improved because information about the community structure acts as a proxy for micro-environmental conditions, for which direct data are not available.

### Incorporating community structure in SDMs

SDM predictions of species distribution and community structure improved when information on community structure was included. Several of the species for which community structure information improved SDMs have specific habitat requirements. *Corynephorus canescens* requires bare and sandy soils [57], *Salix atrocinerea* occupies river banks and permanently wet soils [58], and *Quercus canariensis* occupies shaded and humid canyons [59]. By contrast, species for which community structure information does not improve SDMs often have wide distributions in the Mediterranean region (*Quercus ilex* [59]) or are highly generalist and exhibit invasive behaviour in non-native regions (*Brachypodium sylvaticum*, *Hedera helix* [60–62]) (S2 Table). Therefore, the micro-environmental data added by community structure might be especially informative for species with restrictive ecological requirements, and less relevant for more generalist species.

Information about micro-climatic conditions is rarely available across large spatial extents such as the Iberian Peninsula (though climate data can be downscaled [63]). However, information about the community structure of coexisting plant species is often available across large extents, and can act as a substitute for micro-climatic information that cannot be otherwise included in SDMs.

### Using traits to explore ecological processes underlying abundance covariance between species

We caution against simply assuming that co-occurrence patterns reflect biotic interactions. Instead, we suggest that asking whether associations occur between species with similar or dissimilar ecological requirements can provide insight into the predominance of biotic interactions and environmental filtering. Community assembly theory suggests that biotic interactions and environmental filtering can affect the distribution of trait values within communities (i.e. by permitting different sets of species to co-exist). Environmental filtering leads to coexisting species having similar traits as a result of shared ecological tolerances [64,65]. However, non-consumptive interactions like competition and facilitation can have varying effects on traits, depending on the traits and details of the interaction. For example, most studies focusing on competition have been based on the common assumption that species with similar ecological strategies compete more intensely for resources than species with different strategies [66] resulting in co-existing species having different traits. On the other hand, competition can magnify the effects of environmental filtering by causing species with similar traits to co-occur. For example, competition for light in shaded environments can lead to species with the same light-adaptation traits outcompeting species with different traits [67]. Positive biotic interactions such as facilitation (i.e. one species directly promotes the presence of another [68]) can result in a positive association between ecologically dissimilar species, because this ecological process is frequent between phylogenetically distant plant species [35,69,70]. Alternatively, facilitative interactions driven by shared mutualists such as pollinators, can result in a positive association between plants with similar floral traits, as similar flowers enhance the attraction of shared pollinators [71]. The potential for different trait co-occurrence patterns to arise from the same type of biotic interactions therefore adds complexity to the interpretation of trait data to explain species co-occurrence. However, we suggest that considering traits appropriate to the situation can be highly informative when interpreting causes of co-occurrence patterns.

**Fig 4:**
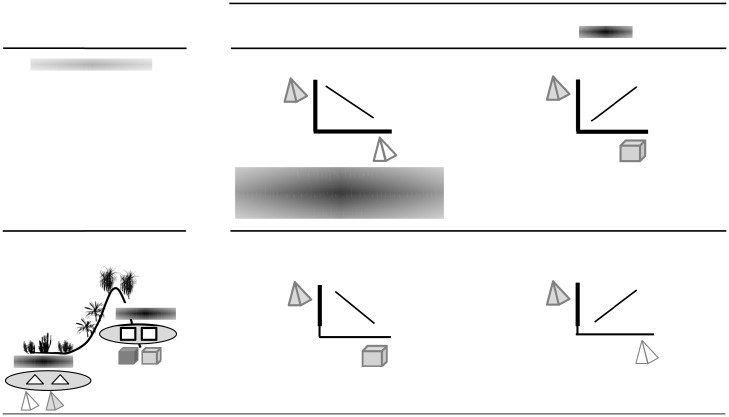
Expected covariance between species involved in biotic interactions and environmental filtering. The combination of 3-d shapes and colors represent four different species. Species with similar requirements (syndromes) are represented by the same shape (pyramids: Tertiary (T), cubes: Quaternary(Q)), but distinct colors. Environmental filters are represented as grey ellipses in which only species with certain traits can survive (e.g. moist and shaded environments on north facing slopes where species with a tertiary syndrome can survive, or sunny environments on south facing slopes where quaternary species can survive: the 3-d shapes must match the shape of the ellipse). In the case of negative abundance covariance, competition is expected to be more intense between species with similar traits and ecological requirements resulting in spatial segregation between species with similar requirements and traits, while environmental filtering will result in spatial segregation between species with dissimilar requirements and traits. In the case of positive abundance covariance, facilitation promotes the co-occurrence between species with dissimilar requirements and traits, while habitat filtering results in the co-occurrence of species with similar requirements and traits.

The tendency for Tertiary species (which are associated with humid, shaded areas) to co-occur, suggests that their presence can provide information about micro-environmental conditions, specifically shade and soil moisture (Fig 4). An alternative explanation could be that the species are facilitating each other’s reproduction by attracting shared pollinators [71]. However, only two of the 14 morphological and functional traits used to define Quaternary and Tertiary syndromes relate to the pollination syndromes [49]. In addition, the plants studied showed neither entomophily or anemophilia, so there was little inter-specific variation in floral morphology. Therefore, although we cannot completely rule out the possibility that facilitation through enhanced attraction of shared pollinators underlies the co-occurrence of ecologically similar plant species in our study, we consider it unlikely.

Although environmental filtering appears to explain the co-occurrence patterns found, environmental filtering would also be expected to result in negative links among species that inhabit different habitat types, with the same frequency as positive links [72,73]. The predominance of positive links in our network (Fig 1 and S3 Table) might be because the study system is defined by the presence of *Quercus suber* which has relatively restricted habitat requirements, resulting in insufficient environmental variation to reveal strong segregation between Quaternary and Tertiary species. The predominance of positive species associations has been also reported in other studies of species associations [25,74–76].

Although our results suggest that environmental filtering drives species associations, plant-plant facilitation (positive interactions) between species with Quaternary and Tertiary syndromes is known to have played a crucial role in the persistence of the latter [46]. It may be possible to detect facilitation at an even finer spatial resolution than we studied. Quaternary-Tertiary facilitation may often take the form of improved seedling recruitment under adult plants, which might be apparent if networks are created using plant abundance data on the scale of a few meters. The ecological processes captured by network inference may therefore depend on the spatial resolution of the analysis.

In conclusion, we show how BNIs can improve understanding of species distributions, and how this could improve SDMs. The network structure provided by the BNI can be combined with ecological trait data to explore potential processes underlying species associations. However, these interpretations should be made cautiously, given that different mechanisms could result in similar patterns. Taking this into account, we consider it likely that species abundance in Mediterranean woody plant communities, at the resolution studied, arise from micro-environmental associations that are rarely detectable using standard SDM approaches.

## Supporting information

S1 Appendix Further detailed information about plot characterization, environmental variables, climatic variables, geological information, network inference, variables selection, and “Env+Bio” and”Env” comparison. (DOC)

S1 Fig Sampling area and location of the plots used in the study. (EPS)

S1 Table Sp. syndrome. Names of the species and code used for each of them, syndrome assigned and reference supporting the assignment to that syndrome.
(XLSX)

S2 Table Env+Bio and Env models. Summary of the SDMs constructed used for each species. Spearman correlation between their predictions and the observed abundance for each species, considering the validated plots (“validate”) and those used in the analysis (“test”), the deviance and deviance explained for each model, and the difference between the correlation with the observed data obtained using the “Env+Bio” and “Env” model for each species.
(XLSX)

S3 Table Links. Summary for all the significant links inferred between species. Species involved (from: parent node, to: children node), strength and direction of the association based on the number of times that the link appears in the resampled networks, sign and significance of the sign based on the Jonckheere trend test and the syndrome code for the interspecific association.
(XLSX)
