# Correction: Community structure informs species geographic distributions

**DOI:** 10.1371/journal.pone.0200556

**Published:** 2018-07-09

**Authors:** Alicia Montesinos-Navarro, Alba Estrada, Xavier Font, Miguel G. Matias, Catarina Meireles, Manuel Mendoza, Joao P. Honrado, Hari D. Prasad, Joana R. Vicente, Regan Early

Fig 1 and Fig 4 are incorrect. The authors have provided corrected versions of Fig 1 and Fig 4 here.

**Fig 1:**
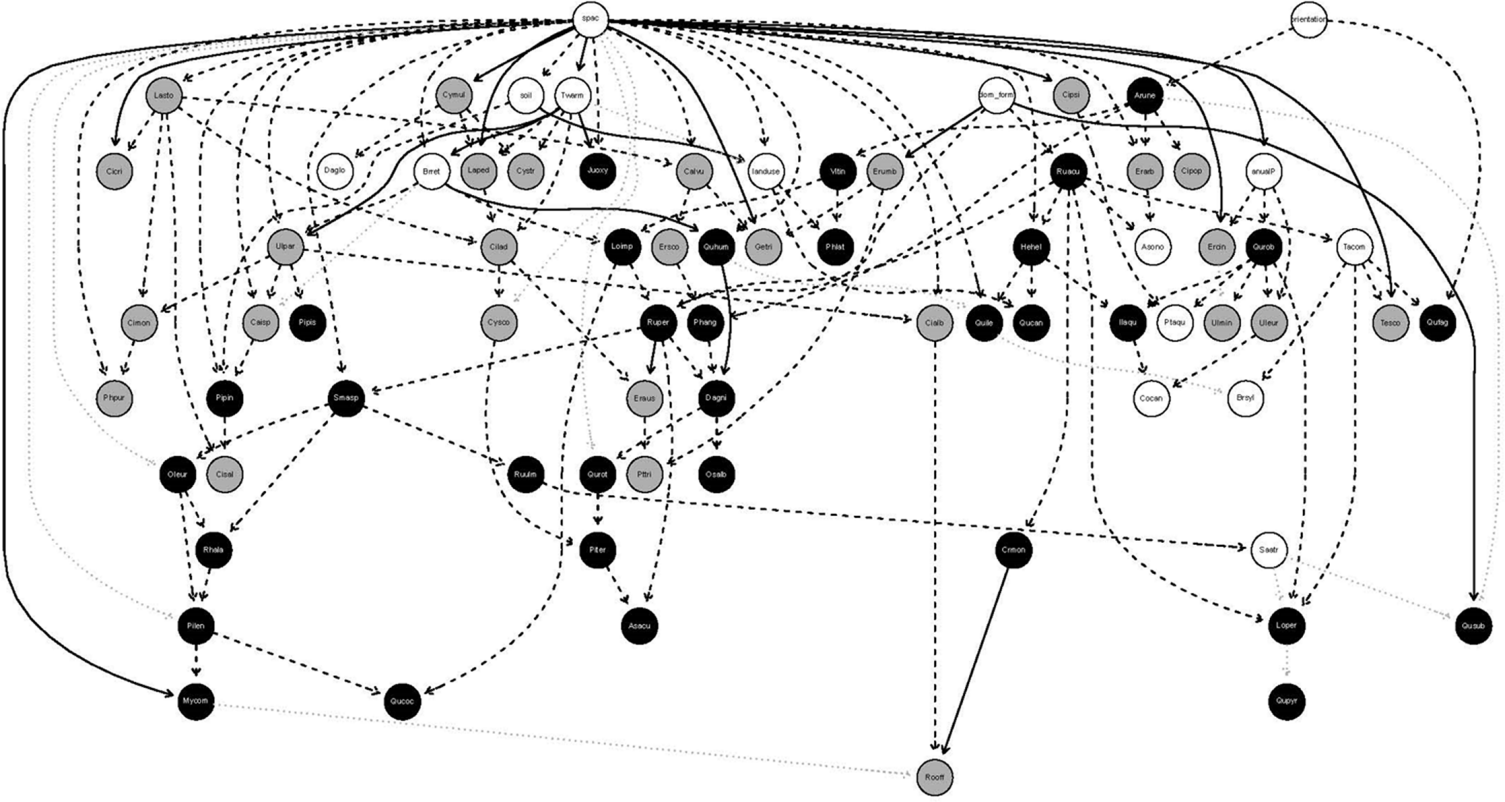
Network structure learned using Bayesian network inference. Only significant links are presented, and grey lines indicating links with no sign was detected. Grey and black circles represent species with a Quaternary and Tertiary syndrome respectively. White circles are either environmental variables (mean temperature in the warmest quarter of the year (Twarm), annual precipitation (anualP), soil types (soil), land use (landuse), orientation (orientation), dominant form (dom_form) and spatial location (spac)) or species with no syndrome associated. Continuous and dashed lines represent negative and positive associations respectively. Complete names for species are provided in the appendix and environmental variable categories in the methods section.

**Fig 4:**
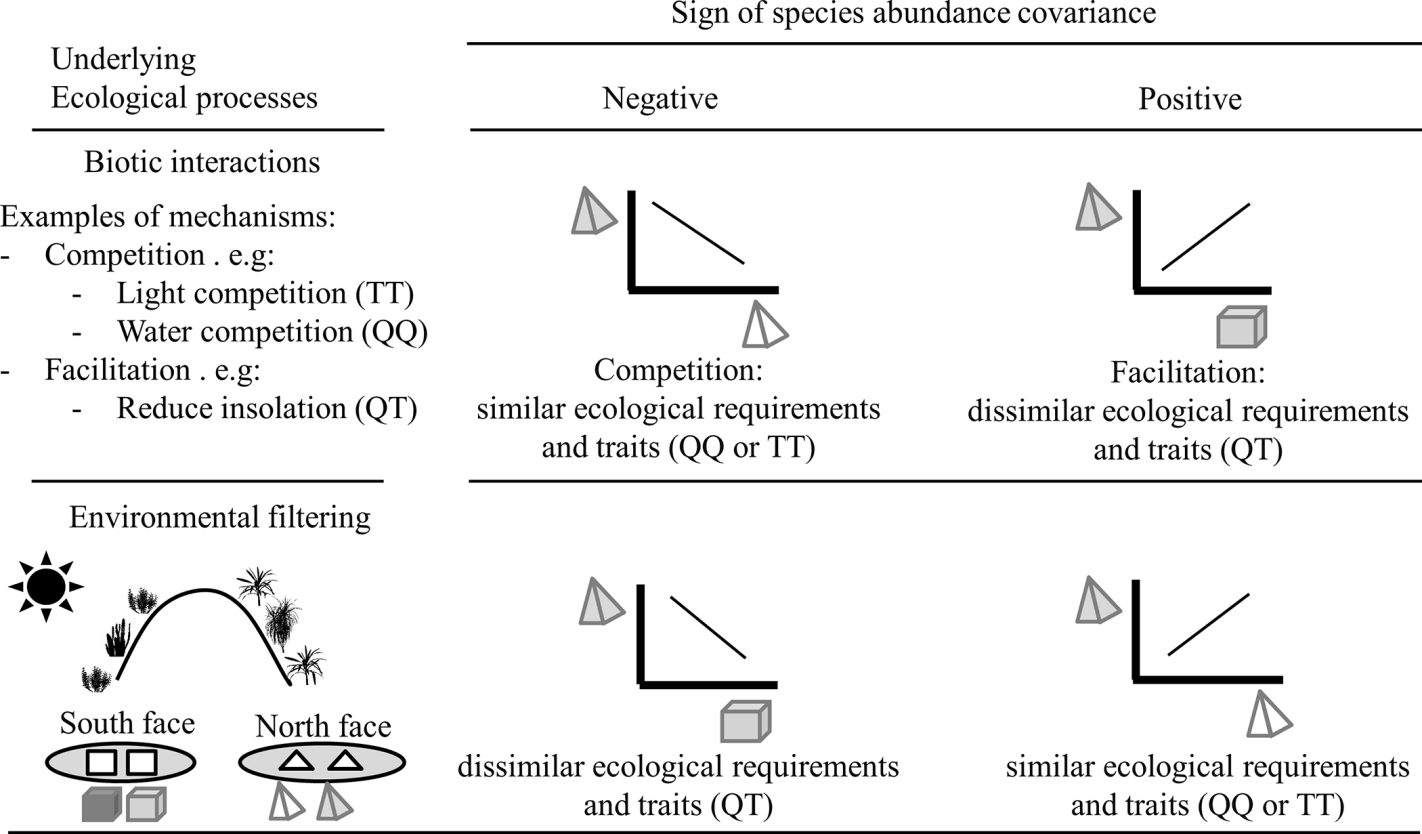
Expected covariance between species involved in biotic interactions and environmental filtering. The combination of 3-d shapes and colors represent four different species. Species with similar requirements (syndromes) are represented by the same shape (pyramids: Tertiary (T), cubes: Quaternary(Q)), but distinct colors. Environmental filters are represented as grey ellipses in which only species with certain traits can survive (e.g. moist and shaded environments on north facing slopes where species with a tertiary syndrome can survive, or sunny environments on south facing slopes where quaternary species can survive: the 3-d shapes must match the shape of the ellipse). In the case of negative abundance covariance, competition is expected to be more intense between species with similar traits and ecological requirements resulting in spatial segregation between species with similar requirements and traits, while environmental filtering will result in spatial segregation between species with dissimilar requirements and traits. In the case of positive abundance covariance, facilitation promotes the co-occurrence between species with dissimilar requirements and traits, while habitat filtering results in the co-occurrence of species with similar requirements and traits.
