# Supplementary material for: Community structure informs species geographic distributions
Source: PLoS One. 2018 May 23;13(5):e0197877. doi: 10.1371/journal.pone.0197877 (PMC5965839; doi:10.1371/journal.pone.0197877)
Supplement: S1 Appendix — (DOC) [file pone.0197877.s001.doc]

METHODS APPENDIX

*Plot characterization*

The plots were sampled following the methods established by Braun-Blanquet et al. (1932) (Braun-Blanquet, Fuller & Conard 1932) in which plots are centrally located in areas with homogenous vegetation canopy and soil. Plot size varies from a few meters to a few thousand meters, as it must be small (or large) enough to adequately characterize the species composition of different vegetation types, but with considerable variation in species richness within plots of a similar size (Fig. 1). Within each plot, a complete species list is recorded, and the ground covered by the vertical projection of the aerial parts of each plant species is recorded using the following percentage classes: 1 (1-5%); 2 (5-25%); 3 (25-50%); 4 (50-75%); and 5 (>75%) (Braun-Blanquet et al. 1932). For simplicity we will refer to this percentage cover as ‘abundance’. Only species with an abundance of at least class 2 Braun-Blanquet’s scale (i.e. that covered on average 15% of the plot area) in at least 1% of the plots were considered for the final species pool. The selected plots were sampled between 1934 and 2013, and have areas ranging from 1 to 3000 m^2^ depending on whether the vegetation sampled was shrubs (small plots) or forests (large plots). This variation in plot area should not influence the relative abundance of each species, as they are expressed as a percentage of the plot area.

*Figure 1. Plant species richness present in plots of different area used in the study.*

*Environmental variables*

All the plots were assigned the environmental data (described below) of the 10 km grid cell within which each of them occurred (n = 444). This was the finest resolution at which all plots could be referenced.

*Climatic variables*

The model setup, the quality of the data and its validation has been previously reported (Prasad et al. 2014; Prasad & Srinivas 2015). Climatic records from 1961 to 1990 of mean temperature (°C) and total precipitation per month (mm per day ×30) were used to calculate several bioclimatic variables: mean temperature of wettest quarter, mean temperature of driest quarter, mean temperature of warmest quarter, mean temperature of coldest quarter, annual precipitation, precipitation of wettest quarter, precipitation of driest quarter, precipitation of warmest quarter, precipitation of coldest quarter. Covariance occurred between all temperature-related variables (Pearson correlation coefficient between any pair of variables r > 0.3, p < 0.001) and within precipitation-related variables (r > 0.5, p < 0.001). We therefore selected the most general precipitation-related variable (mean annual precipitation) and a single temperature-related variable that had the lowest correlation coefficient with mean annual precipitation (mean temperature of warmest quarter).

*Geological information*

The broadest lithology and fragmentability categories provided in this database were used to group the geological types in our study area into 7 broad categories: igneous rock, fragmental igneous material, foliated metamorphic, metamorphic rock, clast sediment, sedimentary rock and unknown.

*Network inference*

In order to choose the best settings to infer the Bayesian networks (BN), we performed 60 different searches resulting from the combination of 10 categorizations of continuous variables × 2 searching algorithms (Hill-climbing and Tabu) × 3 different numbers of bootstrap replicates (100, 500, 1000). The settings using the “Tabu” algorithm with 500 bootstrap replicates resulted in the highest Bayesian Dirichlet equivalent score proposed by Heckerman (1995). The “Tabu” algorithm makes an stochastic search following steps: (i) It starts with an empty directed acyclic graph (DAG) (i.e. without any edge). (ii) One edge is added, deleted or reversed at a time. (iii) The prior DAG is compared against the data, and the score used to assess the fit of the data to the graph is calculated. (iv) The change performed is accepted if the score increases. “Tabu” algorithm permits movements that worsen the score only if no improving movement is available, and Tabu introduces prohibitions to prevent searches from coming back to previously-visited solutions (Glover 1986). (v) A new DAG is suggested based on the changes accepted (or not) in the previous DAG. (vi) These steps are iterated until the score can no longer be improved. The aim of this process is to maximize the probability of finding a given combination of all the variables, given the database. Thus, considering three variables x_1_, x_2_ and x_3_, during the learning process a link between x_1_ and x_3_ would be avoided if p (x_3_| x_1_,x_2_) = p (x_3_ | x_2_), and this decision will be made based on whether the data support the equality or not. These analyses were performed using the functions “boot.strength” and “averaged.network”, implemented in the “bnlearn” package in R, specifying "bde" as an algorithm attribute (Scutari 2010).

Multinomial BNs require continuous environmental variables to be categorized. In order to choose the best categorization, we repeated the previous steps using ten different categorizations of the environmental variables. The best network score was obtained considering 4 levels of species’ relative abundance (mean value: 0%, 7% [range: Braun-Blanquet’s classes 1-2], 50% [3-4], 80% [5]), three levels of mean temperature in the warmest quarter of the year ( [< 14 °C], [14-19 °C] and [> 19 °C]), four levels of annual precipitation (mm per year) ([< 150 mm], [150-449 mm], [450-900 mm], [> 900 mm]), three levels of geological types (sediments [clastic or foliated], mix, rocks [sedimentary, metamorphic, igneous]), two levels of land-use (mix, forest), three levels of orientation (South [SE, SW], East-West [E,W], and North [NE, NW]), three levels of dominant form (shrub, mix, forest) and five levels of spatial location (Military grid reference system (MGRS): 29S, 29T, 30S, 30T, 31T) (Fig.2). For some analyses (see below) the environmental categories need to be ordered to give a sign to the links in which they were involved. The order used was the same in which we listed the levels of each variable above. The order reflects positive ecological gradients from south (drier conditions) to north (wetter conditions), from more permeable rock (sediments) to more solid rock (igneous rocks), from more disturbed (mix of agriculture and forest-shrubs) to less disturbed (dominant forest-shrubs) areas and from communities where the dominant growth form is shrubs, a shrub-forest mix, or forest.

*Performance of network inference as a method for variable selection*

In order to assess the performance of network inference in selecting variables, we test whether the inclusion of the biotic variables identified by BNI result in more accurate predictions than models including randomly selected variables. To do so, we also performed the same GAM analyses described in the methods section using randomly selected variables, instead of those selected by BNI. For each plant species, we randomly selected the same number of abiotic and biotic variables that were contained in its original model. We first maintained the abiotic variables and only replaced the biotic variables with other randomly selected biotic variables, and second we replaced both biotic and abiotic variables with randomly selected variables of any type. In both cases the results are the same. For each plant species, we performed the GAM using the randomly selected variables and calculated the R^2^ spearman correlation coefficient between the observed and predicted abundance. For each species, we repeated this process 1000 times, choosing each time a different random selection of variables. Then, we quantified the percentage of times (out of the 1000) that our original result (using variables selected by BNI) had a R^2^ higher than the random models for each species. For 44 out of the 68 species (64%), our original model had a R^2^ higher than 90% of the 1000 random models. We also found a positive significant correlation between the R^2^ of our original models for each plant species and the percentage of times that each model performed better than the 1000 models based on a randomly selected set of variables (Estimate = 1.34, SE = 0.06, p-value= <0.0001). This suggests that the 22 species in which our original model does not predict the observed abundances better than the random models were those for which the original prediction was not particularly accurate.

*Comparing “Env+Bio” and ”Env” variables in SDMs*

The SDMs generated with our *Env+Bio* and *Env* approaches do not necessarily have the same sets of environmental variables, and thus are not nested within each other. However, we use Akaike Information Criterion (AIC) as the measure of relative quality of each model. AIC deals with the trade-off between the goodness of fit of each model and its complexity (number of parameters) penalizing the models with more variables by its mathematical definition:

AIC = 2k-2ln(L)

where, L is the maximized value of the likelihood function of the model (i.e. probability of the data given the parameters that describe the model)), and K the number of parameters to be estimated (which will increase with the number of variables included in the model). AIC, unlike other measurements of relative quality of each model as for example likelihood-ratio test, is not used for formal hypothesis testing, but for informal comparisons of models with differing numbers of parameters. The penalty term in the expression for AIC is what allows this comparison. As it is not used for formal hypothesis testing, no assumptions are made about the functional form of the asymptotic distribution of the differences between the AIC of two non-nested models, as the difference between two AICs is not treated as a test statistic, thus the use of AIC is not restricted only to nested models as the likelihood-ratio test (Burnham & Anderson 2002).

REFERENCES

Braun-Blanquet, J., Fuller, G.D. & Conard, H.S. (1932) Plant Sociology. The Study of Plant Communities. McGraw-Hill Book Co., Inc., New York and London.Burnham, K. P.; Anderson, D. R. (2002), Model Selection and Multimodel Inference: A Practical Information-Theoretic Approach (2nd ed.), Springer-Verlag.
Glover, F. (1986) Future paths for integer programming and links to artificial intelligence. Computers and Operations Research, 13, 533–549.Heckerman, D., Geiger, D. & Chickering, D.M. (1995) Learning Bayesian networks: The combination of knowledge and statistical data. Machine learning, 20, 197–243.
Prasad, D.H., Salgado, R., Perdigao, J. & Challa, V.S. (2014) A Regional Climate Simulation Study Using WRF-ARW Model over Europe and Evaluation for Extreme Temperature Weather Events. International Journal of Atmospheric Sciences, 2014.
Prasad, H.D. & Srinivas, C.V. (2015) A Study of Precipitation Climatology and Its Variability over Europe Using an Advanced Regional Model (WRF). American Journal of Climate Change, 4, 22.Scutari, M. 2010. Learning Bayesian Networks with the "bnlearn" R Package. Journal of Statistical Software 35: i03.
